# Intratumoral distribution of anisokaryosis in canine cutaneous mast cell tumors

**DOI:** 10.1177/03009858261465447

**Published:** 2026-07-12

**Authors:** Christof A. Bertram, Frauke Wilm, Eda Parlak, Taryn A. Donovan, Hannah Janout, Pompei Bolfa, Michael J. Dark, Andrea Fuchs-Baumgartinger, Andrea Klang, Robert Klopfleisch, Barbara Richter, Marc Aubreville, Stephan M. Winkler, Matti Kiupel, Alexander Bartel

**Affiliations:** 1 University of Veterinary Medicine Vienna, Vienna, Austria; 2 Friedrich-Alexander-Universität Erlangen-Nürnberg, Erlangen, Germany; 3 Mira Vision Microscopy GmbH, Göppingen, Germany; 4 The Schwarzman Animal Medical Center, New York, NY; 5 University of Applied Sciences Upper Austria, Hagenberg, Austria; 6 Johannes Kepler University Linz, Linz, Austria; 7 Ross University School of Veterinary Medicine, Basseterre, Saint Kitts and Nevis; 8 University of Florida, Gainesville, FL; 9 Freie Universität Berlin, Berlin, Germany; 10 Hochschule Flensburg, Flensburg, Germany; 11 Michigan State University, Lansing, MI

**Keywords:** anisokaryosis, canine, deep learning, dog, heterogeneity, mast cell tumor, morphometry, neoplasia, nuclear pleomorphism

## Abstract

Automated measurements of anisokaryosis in canine cutaneous mast cell tumors (ccMCTs) have been shown to be predictive of survival, but questions remain regarding the intratumoral distribution of anisokaryosis. Whole-slide images of 96 ccMCTs were analyzed with a deep learning-based segmentation algorithm to quantify anisokaryosis using the standard deviation (SD) of the nuclear area. In 35/96 cases, >5% of the non-overlapping 256 × 256 µm^2^ regions were hotspots (SD ≥11.5 µm^2^). Regions selected by 7 pathologists within these 35 cases matched hotspots in 32% of the instances. Outcome analysis (tumor-related death) based on single tumor regions yielded an area under the curve (AUC) of 0.901 for pathologist-selected hotspots, falling between random region selection (AUC: 0.862) and 90th-percentile targeted selection (AUC: 0.956). Whole-slide analysis of the hotspot proportion predicted survival with an AUC of 0.956, with 20% of hotspots as a prognostically meaningful threshold. Whereas pathologists-selected tumor regions are prognostically meaningful for nuclear morphometry, whole-slide analysis may provide additional prognostic information.

Various nuclear features in canine cutaneous mast cell tumors (ccMCTs) have been shown to be strong predictors of the biological behavior.^5,11,17^ These nuclear features are traditionally determined through time-efficient visual estimations by pathologists. For example, the 2011 grading system for ccMCT^12^ classifies cases based on anisokaryosis (also referred to as karyomegaly) into 2 groups: present and absent. However, visual estimates are prone to low inter-observer reproducibility in ccMCT^11^ and other tumor types.^9,16^ A more reproducible, but also methodologically complex approach is nuclear morphometry,^6,9,11,16^ which has been recently evaluated for ccMCT, either through 3-dimensional mean volume calculations based on stereological concepts^6,7^ or 2-dimensional area measurements.^10,13^

Manual morphometry methods require an average of 10–17.5 minutes per case and are not routinely feasible.^6,11,16^ In contrast, automated morphometry offers improved efficiency and is facilitated by the recent advancements in computational pathology. Notably, the use of deep learning-based algorithms provides high analytic accuracy and thereby enables the extraction of meaningful data from larger tumor areas.^1,9,11^

Whereas morphometric analyses have typically been performed on small, randomly or arbitrarily selected tumor regions,^6,7,9^ a study by Haghofer et al^11^ demonstrated intratumoral variability in morphometric measurements when comparing 5 regions per ccMCT. For other prognostic parameters, such as the mitotic count^4,15^ and the Ki67 index,^18^ the areas with the highest density (ie, hotspots) are considered most prognostically relevant. This raises the question of whether this also applies to anisokaryosis. Furthermore, the study by Haghofer et al^11^ suggested that tumors with high degrees of anisokaryosis in multiple areas are associated with an increased risk of tumor-related death. This warrants a more in-depth analysis of the distribution of anisokaryosis across entire tumor sections and the prognostic value of the proportion of hotspot locations per tumor section.

By computing anisokaryosis across entire tumor sections of ccMCT, this study has 4 objectives:

1. Describe the spatial intratumoral distribution of anisokaryosis in ccMCT.
2. Explore whether tumor regions with relatively high degrees of anisokaryosis are more meaningful for prognostication.
3. Assess the ability of pathologists to subjectively identify tumor regions with the highest degrees of anisokaryosis.
4. Evaluate the prognostic relevance of the proportion of hotspot regions.

For this study, the same population as used by Haghofer et al^11^ was utilized, consisting of 96 cases of ccMCT with 1 whole-slide image (WSI) per case. These cases were not used for model development; instead, their use was restricted to the validation of the algorithms’ prognostic ability (application data set). WSIs at a single focus plane (resolution: 0.25 µm/pixel) were created from hematoxylin and eosin-stained histological slides using the Pannoramic Scan II (3DHistech, Hungary) scanner. At least 12 months of follow-up data were obtained through surveys sent to the submitting veterinarians. Of the 96 patients, 13 died from mast cell tumor-related death and 83 were alive for at least 12 months post-ccMCT diagnosis. Further patient characteristics can be found in the previous publication.^11^

WSIs were analyzed by the deep learning-based nuclear segmentation algorithm from Haghofer et al^11^ to predict the nuclear areas of all tumor cells. The high performance of this model has been previously demonstrated, including a Dice score of 0.785 on the test data set.^11^ For each segmented cell nucleus, its center coordinate and its measured area in µm^2^ was determined. Algorithmically predicted nuclei were afterwards restricted to meaningful tumor regions by 2 exclusion steps. First, the tumor outline and large non-neoplastic tissues within the tumor (eg, necrotic areas and hair follicles) were manually delineated by 1 student at 10× magnification using the polygon tool of the annotation software SlideRunner (Version 2.0.0).^2^ When consecutive sections of the same block were present on the slide, only 1 section was annotated. All annotations were reviewed and approved by a board-certified pathologist (CAB). The annotated tumor areas were subdivided into non-overlapping regions, each with a size of 1024 × 1024 pixel (256 × 256 µm^2^). The size of tumor regions was determined by balancing the total number of regions per case (to allow a high-resolution determination of intratumoral distribution) and the total number of tumor cells per region (to achieve a large enough sample size for reliable calculations of anisokaryosis per region). The center coordinate of each nucleus was matched with the coordinates of the tumor regions, and all nuclei outside the tumor area were excluded from analysis. Second, the number of predicted neoplastic nuclei per tumor region was calculated, and regions with less than 25 nuclei were excluded, since a low number of nuclei does not allow reliable calculations of anisokaryosis. The study adheres to the *Veterinary Pathology* artificial intelligence-based automated image analysis reporting guidelines; the completed 9-point checklist is provided as Supplemental Table S3.

To evaluate the ability of 7 pathologists to identify regions with the highest degree of anisokaryosis and to determine whether these regions were suitable for algorithmic morphometry, we conducted an experiment using the annotation software EXACT (GitHub master branch commit 80a5ddc, retrieved in September 2024).^14^ Participants were asked to set a bounding box annotation with the fixed size of 1024 × 1024 pixels in the center of their preferred tumor region with 1 region per pathologist in each ccMCT. Participants were blinded to the algorithmic predictions, the results of other participants, and the patient outcome. The regions selected by the pathologists were matched with the above defined center coordinates of the cell nuclei.

Statistical analysis and data management were performed using R version 4.4.1 (R Foundation, Vienna, Austria). For the included image regions, the standard deviation (SD) of the area of the algorithmically predicted objects (mostly neoplastic nuclei) was calculated, and the frequency distribution across all regions per WSI was determined. A tumor region was considered to be an anisokaryosis hotspot if it had an SD of nuclear area of ≥11.5 µm^2^. This threshold has been previously shown to be specific for tumor-related death (sensitivity: 53.8%, specificity: 100%).^11^ The intratumoral proportion of hotspots per tumor was calculated by dividing the number of hotspot regions by all regions. The prognostic ability to predict tumor-specific death was calculated by Kaplan-Meier curves with log-rank test and area under the curve (AUC) along with 95% confidence intervals (CIs). To determine the dependency of region selection on prognostic value, a simulation with random region selection in each WSI with 5000 iterations was used. The simulation was repeated, limiting the random selection to regions above a certain percentile of the SD of nuclear area. This was done for all percentiles up to 90% in 1% steps, which imitates a targeted hotspot selection approach with increasing specificity. For every percentile, the AUC across all tumor cases was calculated.

Each WSI included 21 to 4951 tumor regions (average: 1608), with each case containing a median of 42–255 (average: 124) detected neoplastic nuclei per region. The intratumoral distribution of the SD of the nuclear area is shown in Fig. 1 and Supplemental Figures S1–S7. Most of the 96 cases had an overall low degree of anisokaryosis, with 14 cases having all tumor regions below the threshold of ≥11.5 µm^2^ and 47 cases having <5% of regions above the threshold. The remaining 35 cases included ≥5% hotspot regions (with a median of 18.4% of regions being hotspots), but only 6 of these cases exhibited anisokaryosis hotspots in the majority (>50%) of the regions.

**Figure 1.**
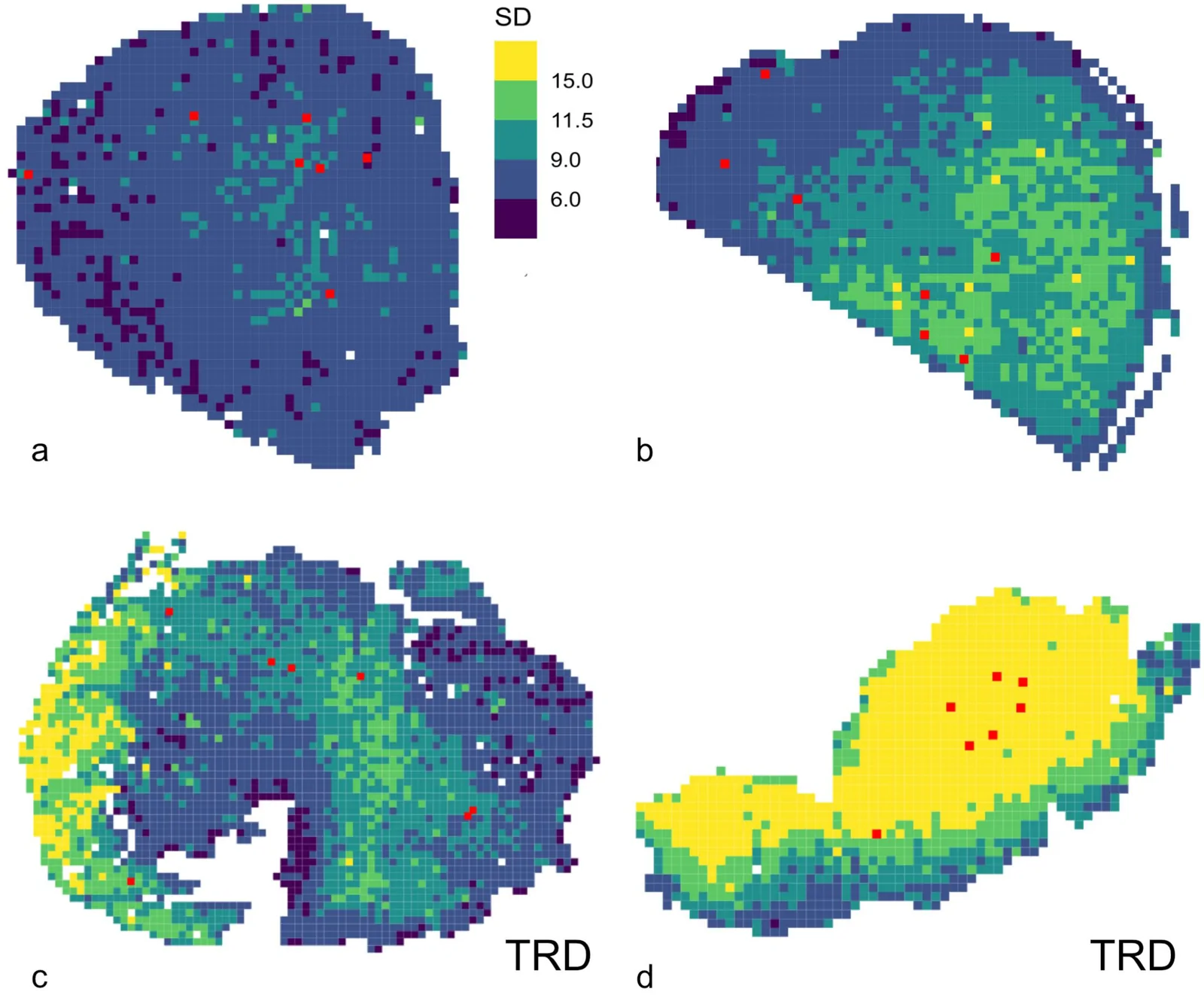
Heatmaps of the anisokaryosis (standard deviation, SD, of nuclear area measured by algorithmic morphometry) from whole-slide images of canine cutaneous mast cell tumors (ccMCT). The tumors are divided into regions, which are classified into 5 degrees of anisokaryosis: dark purple (SD < 6.0 µm^2^), dark blue (SD = 6–8.99 µm^2^), teal green (SD = 9–11.49 µm^2^), yellow-green (SD = 11.5–14.99 µm^2^), and yellow (SD ≥15 µm^2^) using the same thresholds for all images. The red regions represent the areas selected by the 7 pathologists. (a) ccMCT with an overall low degree of anisokaryosis (<0.01% regions above the threshold of SD ≥11.5 µm^2^) from a patient who did not die in relation to the ccMCT. (b) ccMCT with some areas exhibiting marked anisokaryosis (18.2% of the regions are above the threshold of SD ≥11.5 µm^2^) from a patient who did not die in relation to the ccMCT. (c) ccMCT with some areas exhibiting marked anisokaryosis (18.4% of the regions are above the threshold of SD ≥11.5 µm^2^) from a patient with ccMCT-related death (TRD). (d) ccMCT with the majority of the areas exhibiting marked anisokaryosis (76.6% of the regions are above the threshold of SD ≥11.5 µm^2^) from a patient with ccMCT-related death.

Given this variable degree of anisokaryosis and uneven intratumoral distribution of anisokaryosis hotspots, it is important to evaluate which tumor region has the highest prognostic relevance. Based on our simulation, a completely random selection of tumor regions resulted in a baseline AUC of 0.8628 (ie, the median AUC of 5000 iterations with a random region selection for each case) for predicting tumor-related death. Targeted sampling of regions with higher anisokaryosis resulted in an increase in the AUC, reaching 0.9555 when only regions above the 90th percentile of the frequency distribution were considered (Fig. 2a and Table 1).

**Figure 2.**
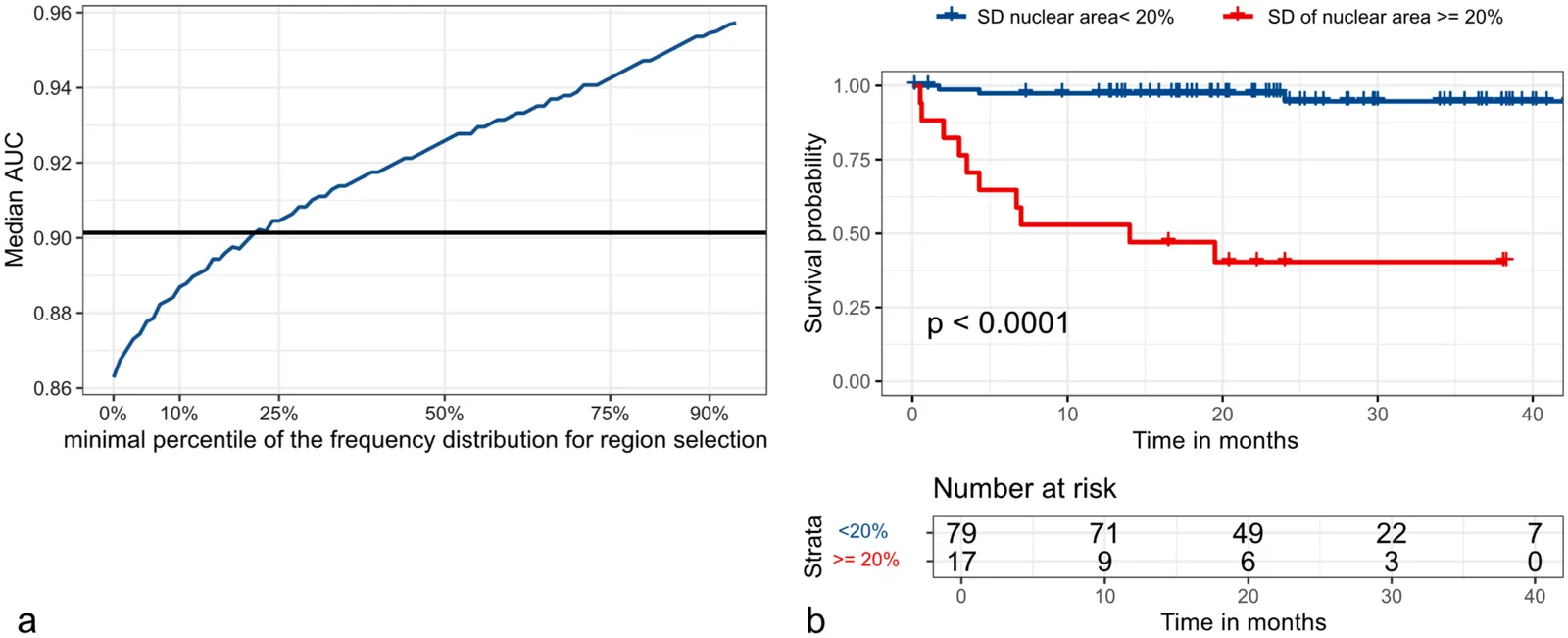
Prognostic value of nuclear morphometry (standard deviation of nuclear area) for analysis of a single tumor region (a) and analysis of entire whole-slide images (b). (a) Simulation of the dependency of the ability to select a hotspot within the entire tumor section on the prognostic value (as measured by the area under the curve, AUC, for tumor-related death; blue curve). For this simulation, tumor regions within a standard deviation of nuclear area above a specific minimal percentile of the frequency distribution (*x*-axis) were randomly selected to calculate the AUC (*y*-axis). This graph shows that the selection of tumor regions with higher degrees of anisokaryosis results in an increase in prognostic relevance. The horizontal black line represents the average AUC for the regions selected by the pathologists. Comparison of the black and blue line shows that the pathologists’ ability to identify intratumoral hotspots is roughly equivalent to a random tumor area above the first quartile of the frequency distribution. (b) Kaplan-Meier curve regarding tumor-specific survival time (in months) for the proportion of hotspot tumor locations (defined as a region with a standard deviation, SD, of nuclear area ≥11.5 µm^2^) within entire whole-slide images. This graph shows that separating patients by a hotspot proportion of <20% and ≥20% is useful in predicting their survival probability (log-rank test, *P* < .0001).

**Table 1.** Dependency of the ability to select a hotspot tumor region on the prognostic value of anisokaryosis evaluation.

Tumor Areas Used for Random Selection	AUC (95% CI)
All tumor regions	0.862 (0.761–0.941)
≥10th percentile	0.887 (0.811–0.951)
≥25th percentile	0.905 (0.845–0.957)
≥50th percentile	0.926 (0.886–0.966)
≥75th percentile	0.943 (0.918–0.968)
≥90th percentile	0.956 (0.943–0.966)

The area under the curve (AUC, tumor-related death) was calculated by randomly selecting a “hotspot” region according to the hotspot definition. This table shows that more targeted hotspot selection, ie, reliably selecting regions with a higher degree of anisokaryosis, would result in an increase in prognostic value.

Abbreviation: CI, confidence interval.

In the real-world experiment, regions selected by the pathologists were often within different locations of the same tumor, demonstrating the large variability in performance of this task between pathologists (Fig. 1). Prediction of tumor-related death based on these selected regions (combining all pathologists) using the algorithmic measurements resulted in an AUC of 0.9013 (Supplemental Table S1). This AUC corresponds to selecting regions above the 25th percentile for anisokaryosis in the simulation (Table 1). A separate evaluation of the cases based on the degree of anisokaryosis revealed that the regions selected by the 7 pathologists matched with a hotspot region (defined based on algorithmic morphometry) in 1.0% (N = 4/396) of instances for cases with <5% hotspots (negligible), in 6% (N = 7/116) of instances for cases with 5%–20% hotspots (low), in 35.6% (N = 26/73) of instances for cases with 20%–50% hotspots (moderate), and in 97.6% (N = 41/42) of instances for cases with >50% hotspot proportion (abundant). Only 93.3% of the pathologist-selected regions could be matched with the analyzed regions due to failure to restrict pathologists’ region selection to the inclusion regions, which was mostly related to images with consecutive sections on the same slide.

Finally, we analyzed the prognostic value of the intratumoral proportion of anisokaryosis hotspots. Most of the 13 cases with tumor-related death had a high proportion of anisokaryosis hotspots (median: 40.5%, range: 11.3%–82.1%), whereas the other 83 cases without tumor-related death had markedly fewer hotspot regions (median: 1.4%, range: 0%–45.6%) (Supplemental Figure S8). The AUC for tumor-related death based on the hotspot proportion was 0.9555 (95% CI: 0.913–0.9981). At a threshold of ≥20% hotspots, tumor-related death could be predicted with a sensitivity of 76.9%, and a specificity of 91.6% (Supplemental Table S2). The survival probability for patients with a hotspot proportion of ≥20% was significantly shorter (Fig. 2b).

As has been shown previously for the mitotic count in ccMCT^4^ and the KI67 index in various canine tumors,^8^ this study demonstrates that the degree of anisokaryosis also exhibits variable intratumoral distribution. For many prognostic parameters in ccMCT, including karyomegaly,^12^ mitotic count,^4^ and Ki67 index,^18^ it is often recommended to evaluate the areas with the highest density/degree. While still unproven for the proliferation markers, our results suggest a slightly improved prognostic discriminative ability of regions with the highest degree of anisokaryosis. These findings should be validated in subsequent studies.

As with the mitotic count,^3^ pathologists have difficulty identifying anisokaryosis hotspot regions. Nevertheless, the morphometry of regions selected by pathologists still resulted in high prognostic value with a substantial increase of the AUC above the random selection baseline. The higher workflow efficiency of using a single pathologist-selected region for automated nuclear morphometry, as opposed to WSI analysis, might justify this approach to overcome computational limitations and reduce processing time. Our data also indicate that it might not be appropriate to find a rare hotspot location in a tumor with an overall low degree of anisokaryosis, as all ccMCTs of this study population with a hotspot proportion below 10% had indolent biological behaviors (ie, no reported tumor-related deaths).

We further demonstrated that cases with a high proportion of anisokaryosis hotspots are associated with poor prognosis. While WSI analysis is computationally expensive, and therefore limited in routine application, our findings suggest that the frequency distribution of anisokaryosis can provide valuable prognostic information. Future studies should evaluate the added value of the prognostic parameter “hotspot proportion” in addition to the computationally cheap analysis of SD of nuclear area in 1 region.

This study has some limitations. Algorithmic predictions were not verified by a pathologist due to the vast amounts of data. Although the study by Haghofer et al^11^ demonstrated a generally high performance, occasional undersegmentation (resulting in an overestimation of nuclear area) may have occurred. Whereas the model was developed to detect only neoplastic mast cells, occasional inclusion of another cell type could have slightly impacted anisokaryosis measurements. Statistical analysis was also complicated by the overall low anisokaryosis observed in most cases. In such cases, region selection has minimal clinical relevance, yet small changes in the region location can markedly influence the percentile ranking within the frequency distribution. This study restricted the experiments to the SD of nuclear area, whereas the frequency distribution of nuclear size may be characterized by other morphometric parameters (such as mean, 90th percentile, or percentage of large nuclei).^11^ The SD of nuclear area has been shown to be one of the prognostically most relevant parameters in ccMCT^11^; however, it is not known if it best represents the pathologists’ approach to estimate anisokaryosis. Thus, a slight discrepancy in the selection criteria by pathologists and our definition of a hotspot might have occurred.

Based on our findings, we can draw the following conclusions for ccMCT:

1. Anisokaryosis has heterogeneous intratumoral distribution, and region selection may influence the prognostic interpretation of borderline cases.
2. Targeted anisokaryosis hotspot selection seems to be prognostically most meaningful.
3. Pathologists have difficulty finding tumor regions with the highest degree of anisokaryosis. However, the pathologist-selected regions still enable an accurate prediction of patient outcome through computerized morphometry.
4. The proportion of anisokaryosis hotspots within a tumor (at a proposed threshold of ≥20%) is a strong prognostic parameter.

## Supplemental Material

sj-pdf-1-vet-10.1177_03009858261465447 – Supplemental material for Intratumoral distribution of anisokaryosis in canine cutaneous mast cell tumors Supplemental material, sj-pdf-1-vet-10.1177_03009858261465447 for Intratumoral distribution of anisokaryosis in canine cutaneous mast cell tumors by Christof A. Bertram, Frauke Wilm, Eda Parlak, Taryn A. Donovan, Hannah Janout, Pompei Bolfa, Michael J. Dark, Andrea Fuchs-Baumgartinger, Andrea Klang, Robert Klopfleisch, Barbara Richter, Marc Aubreville, Stephan M. Winkler, Matti Kiupel and Alexander Bartel in Veterinary Pathology

## References

[1] Alsubaie NM Snead D Rajpoot NM. Tumour nuclear morphometrics predict survival in lung adenocarcinoma. IEEE Access. 2021;9:12322–12331.

[2] Aubreville M Bertram C Klopfleisch R , et al. Sliderunner: a tool for massive cell annotations in whole slide images. In: Maier A Deserno TM Handels H , et al, eds. Bildverarbeitung für die Medizin 2018: Algorithmen-Systeme-Anwendungen. Proceedings des Workshops vom 11. bis 13. März 2018 in Erlangen. Springer; 2018:309–314.

[3] Bertram CA Aubreville M Donovan TA , et al. Computer-assisted mitotic count using a deep learning-based algorithm improves interobserver reproducibility and accuracy. Vet Pathol. 2022;59:211–226. doi:10.1177/03009858211067478 PMC8928234 34965805

[4] Bertram CA Donovan TA Bartel A. Mitotic activity: a systematic literature review of the assessment methodology and prognostic value in canine tumors. Vet Pathol. 2024;61(5):752–764. 10.1177/03009858241239565 PMC11370189 38533804

[5] Camus MS Priest HL Koehler JW , et al. Cytologic criteria for mast cell tumor grading in dogs with evaluation of clinical outcome. Vet Pathol. 2016;53:1117–1123. doi:10.1177/0300985816638721 27034386

[6] Casanova M Branco S Veiga IB , et al. Stereology in grading and prognosis of canine cutaneous mast cell tumors. Vet Pathol. 2021;58:483–490. doi:10.1177/0300985820985138 PMC8064533 33576305

[7] Catarino J Macara A Barros A , et al. Stereological estimation of mean nuclear volume as a prognostic factor in canine subcutaneous mast cell tumors. Vet Pathol. 2025;62:284–292. doi:10.1177/03009858251315094 PMC12014948 39968749

[8] Elena B Barbara B Barbara B , et al. Analysis of Ki67 density maps reveals variation of spatial proliferation patterns in different canine neoplasms. Vet Comp Oncol. 2025;23:642–649. doi:10.1111/vco.70019 40897397

[9] Glahn I Haghofer A Donovan TA , et al. Automated nuclear morphometry: a deep learning approach for prognostication in canine pulmonary carcinoma to enhance reproducibility. Vet Sci. 2024;11:273. doi:10.3390/vetsci11060278 PMC11209399 38922025

[10] Haghofer A Fuchs-Baumgartinger A Lipnik K , et al. Histological classification of canine and feline lymphoma using a modular approach based on deep learning and advanced image processing. Sci Rep. 2023;13:19436. doi:10.1038/s41598-023-46607-w PMC10636139 37945699

[11] Haghofer A Parlak E Bartel A , et al. Nuclear pleomorphism in canine cutaneous mast cell tumors: comparison of reproducibility and prognostic relevance between estimates, manual morphometry, and algorithmic morphometry. Vet Pathol. 2025;62:161–177. doi:10.1177/03009858241295399 PMC11874577 39560067

[12] Kiupel M Webster JD Bailey KL , et al. Proposal of a 2-tier histologic grading system for canine cutaneous mast cell tumors to more accurately predict biological behavior. Vet Pathol. 2011;48:147–155. doi:10.1177/0300985810386469 PMC8369849 21062911

[13] Maiolino P Cataldi M Paciello O , et al. Nucleomorphometric analysis of canine cutaneous mast cell tumours. J Comp Pathol. 2005;133:209–211. doi:10.1016/j.jcpa.2005.02.002 16045919

[14] Marzahl C Aubreville M Bertram CA , et al. EXACT: a collaboration toolset for algorithm-aided annotation of images with annotation version control. Sci Rep. 2021;11:4343. doi:10.1038/s41598-021-83827-4 PMC7902667 33623058

[15] Meuten DJ Moore FM Donovan TA , et al. International guidelines for veterinary tumor pathology: a call to action. Vet Pathol. 2021;58:766–794. doi:10.1177/03009858211013712 34282984

[16] Santos M Correia-Gomes C Santos A , et al. Nuclear pleomorphism: role in grading and prognosis of canine mammary carcinomas. Vet J. 2014;200:426–433. doi:10.1016/j.tvjl.2014.03.019 24745769

[17] de Francisco Strefezzi R Xavier JG Kleeb SR , et al. Nuclear morphometry in cytopathology: a prognostic indicator for canine cutaneous mast cell tumors. J Vet Diagn Invest. 2009;21:821–825. doi:10.1177/104063870902100608 19901282

[18] Webster JD Yuzbasiyan-Gurkan V Miller RA , et al. Cellular proliferation in canine cutaneous mast cell tumors: associations with c-KIT and its role in prognostication. Vet Pathol. 2007;44:298–308. doi:10.1354/vp.44-3-298 17491070

