# Supplementary material for: Intratumoral distribution of anisokaryosis in canine cutaneous mast cell tumors
Source: Vet Pathol. 2026 Jul 12;63(6):1181–6. doi: 10.1177/03009858261465447 (PMC13714219; doi:10.1177/03009858261465447)
Supplement: Supplementary file 1 [file sj-pdf-1-vet-10.1177_03009858261465447.pdf]

Supplemental Materials

Intra-tumoral distribution of anisokaryosis in canine cutaneous mast cell tumors

Christof A. Bertram, Frauke Wilm, Eda Parlak, Taryn A. Donovan, Hannah Janout, Pompei Bolfa, Michael J. Dark, Andrea Fuchs-Baumgartinger, Andrea Klang, Robert Klopfleisch, Barbara Richter, Marc Aubreville, Stephan M. Winkler, Matti Kiupel, Alexander Bartel

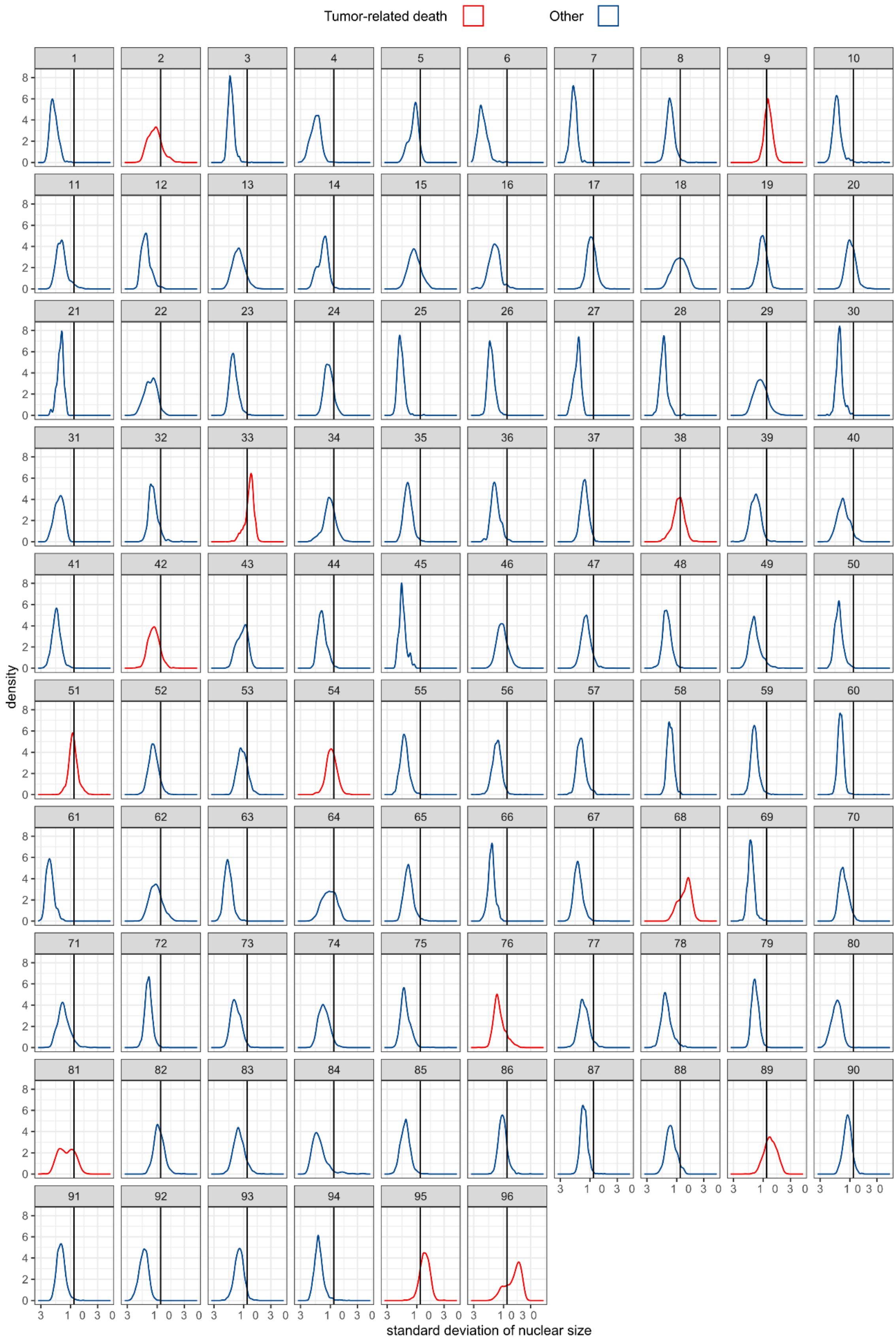

Supplemental Figure S1. Frequency distribution of the standard deviation of nuclear size for all tumor regions within a case (each with one whole-slide image). Cases are categorized into tumor-related death (red lines) and other cases (blue lines). The black vertical line indicates the prognostic threshold of ≥11.5 μm².

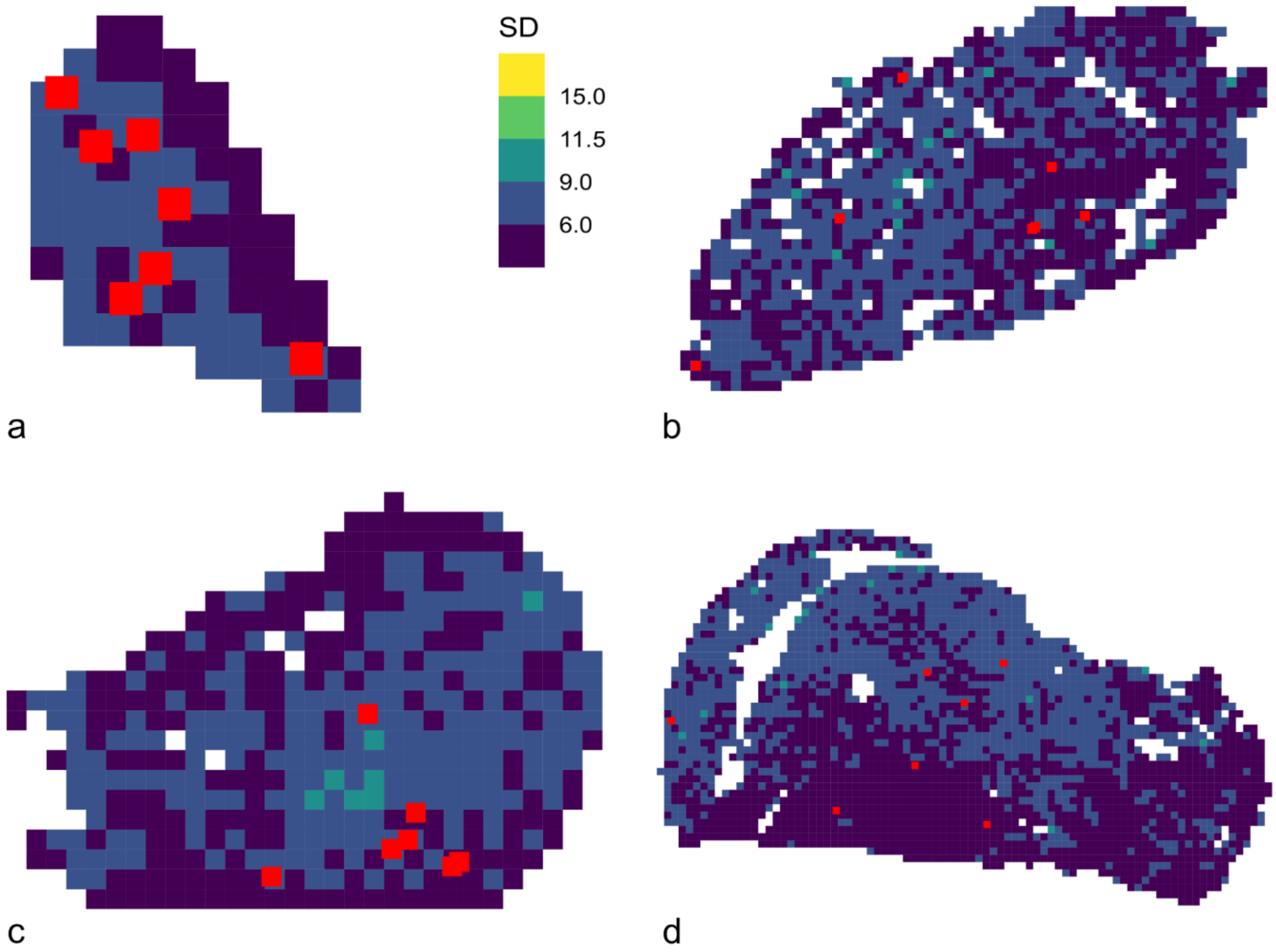

Supplemental Figure S2. Heatmaps of anisokaryosis (standard deviation, SD, of nuclear size measured by algorithmic morphometry) from whole-slide images of canine cutaneous mast cell tumors (ccMCT) with all regions having a SD < 11.5 µm². These patients did not die in relation to the ccMCT. The red patches represent the areas selected by the pathologists.

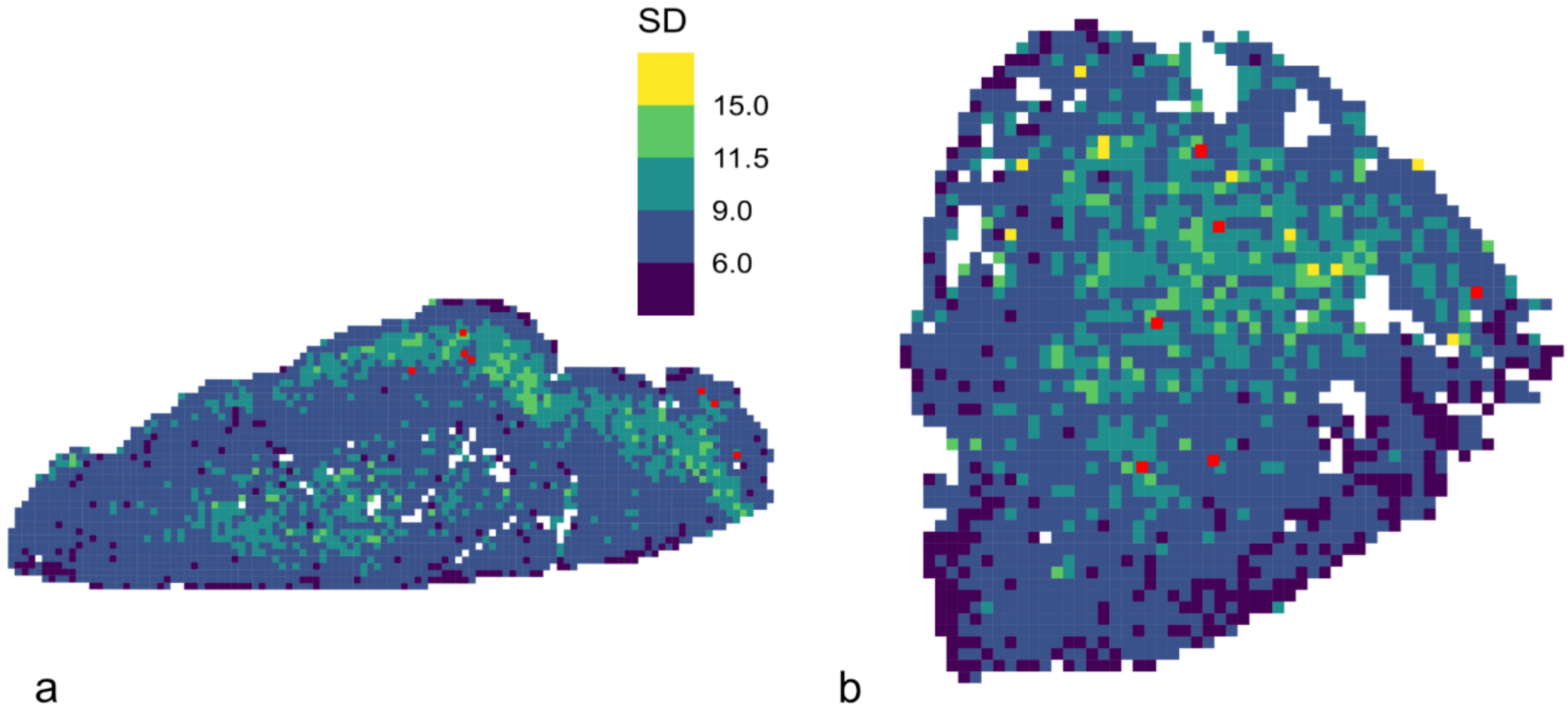

Supplemental Figure S3. Heatmaps of anisokaryosis (standard deviation, SD, of nuclear size measured by algorithmic morphometry) from whole-slide images of canine cutaneous mast cell tumors (ccMCT) with <5% hotspot regions (SD ≥ 11.5 µm²). These patients did not die in relation to the ccMCT. The red patches represent the areas selected by the pathologists. (a) 2.7% hotspots, (b) 4.3% hotspots.

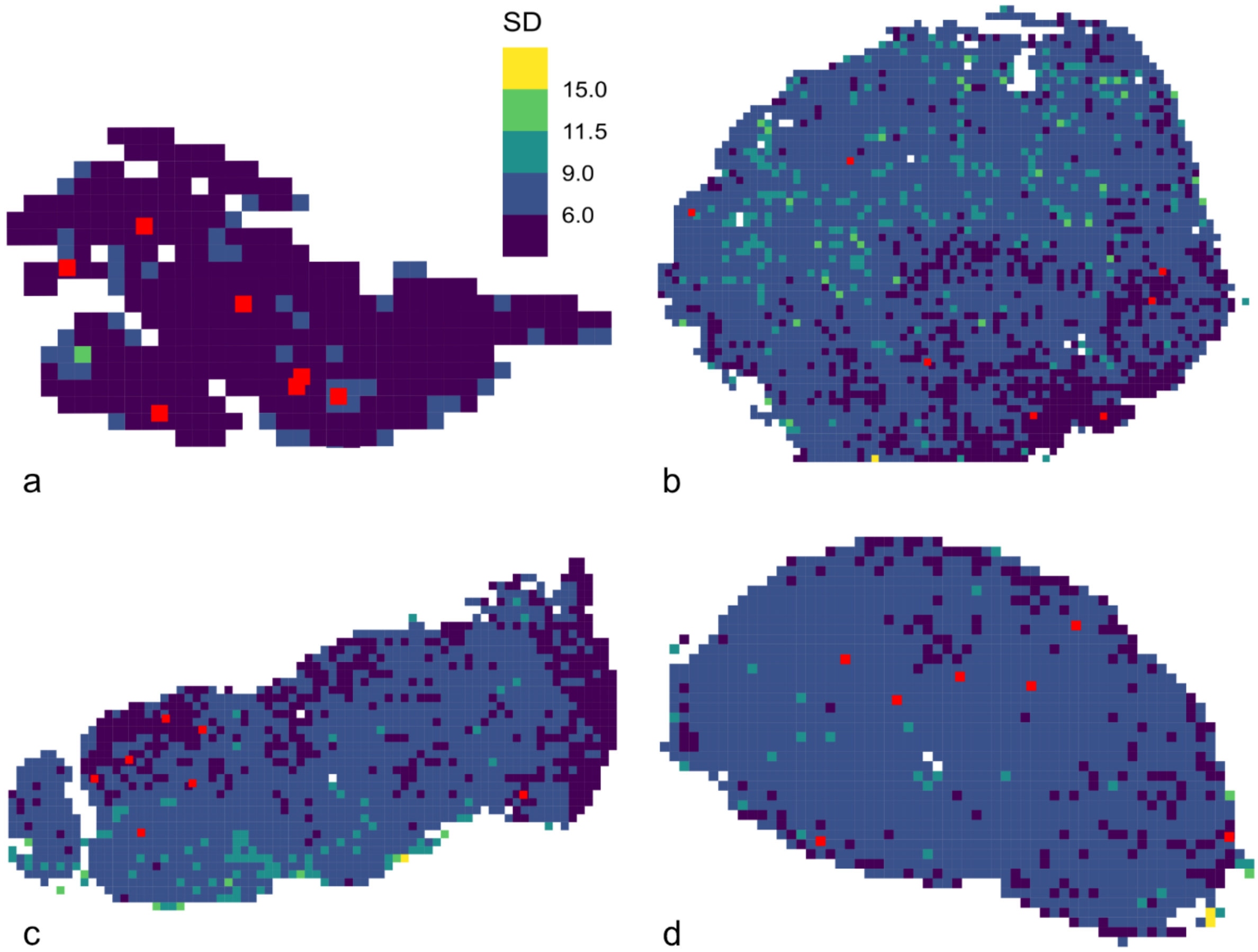

Supplemental Figure S4. Heatmaps of anisokaryosis (standard deviation, SD, of nuclear size measured by algorithmic morphometry) from whole-slide images of canine cutaneous mast cell tumors (ccMCT) with individual hotspot regions (SD ≥ 11.5 µm²). These patients did not die in relation to the ccMCT. The red patches represent the areas selected by the pathologists. (a) 0.3% hotspots, (b) 0.8% hotspots, (c) 0.5% hotspots, (d) 0.2% hotspots. Based on the distribution of these individual hotspots, algorithmic errors due to undersegmentation, detection of non-neoplastic cells, or tissue artifacts in some of these regions could be suspected.

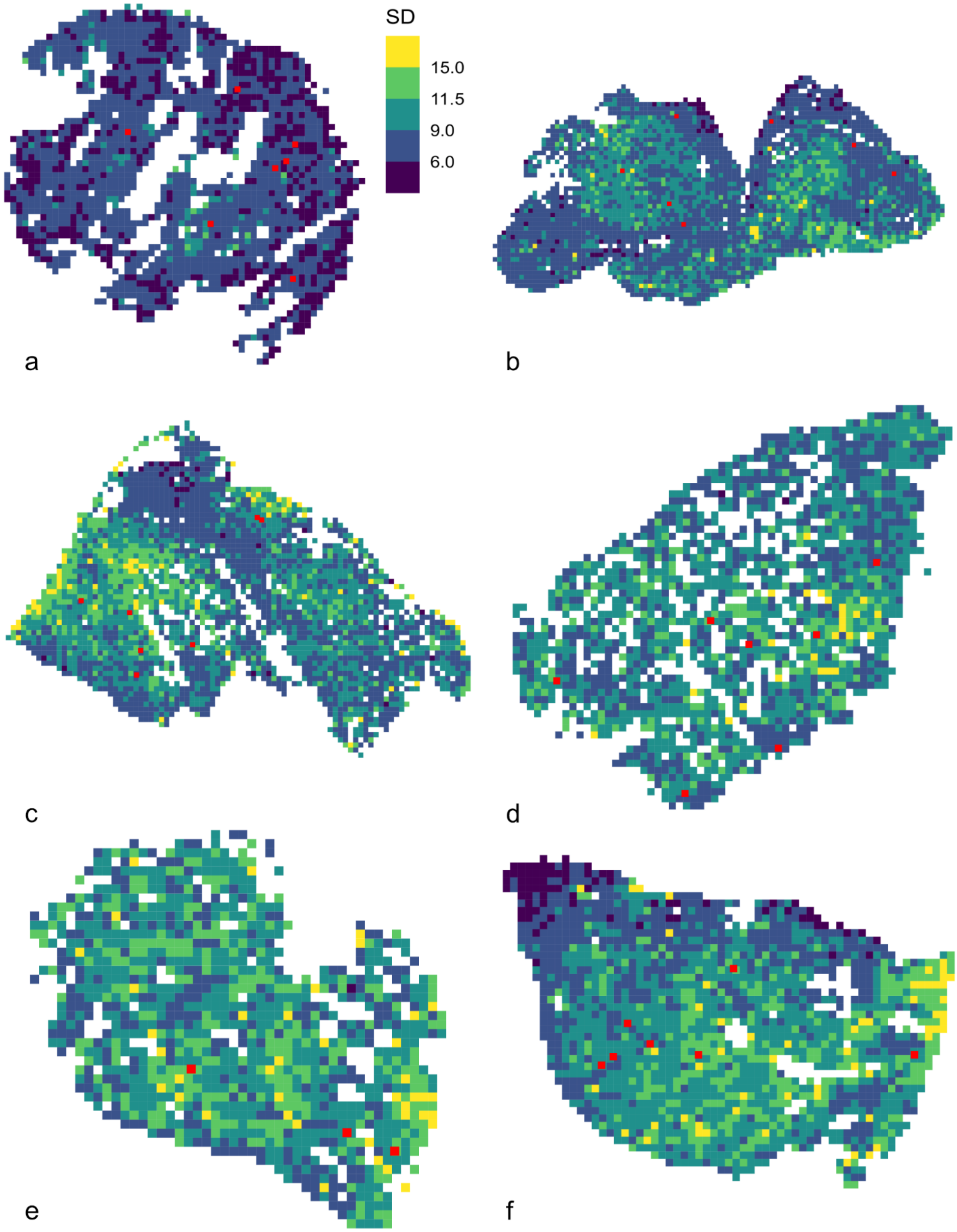

Supplemental Figure S5. Heatmaps of anisokaryosis (standard deviation, SD, of nuclear size measured by algorithmic morphometry) from whole-slide images of canine cutaneous mast cell tumors (ccMCT) with >5% hotspot regions (SD ≥ 11.5 μm^2). These patients did not die in relation to the ccMCT. The red patches represent the areas selected by the pathologists. (a) 6.7% hotspots, (b) 8.1% hotspots, (c) 18.3% hotspots, (d) 14.0% hotspots, (e) 29.0% hotspots, (f) 22.5% hotspots. Some of the individual hotspot regions (such as in (e) and (f)) may represent algorithmic errors due to undersegmentation or detection of non-neoplastic cells.

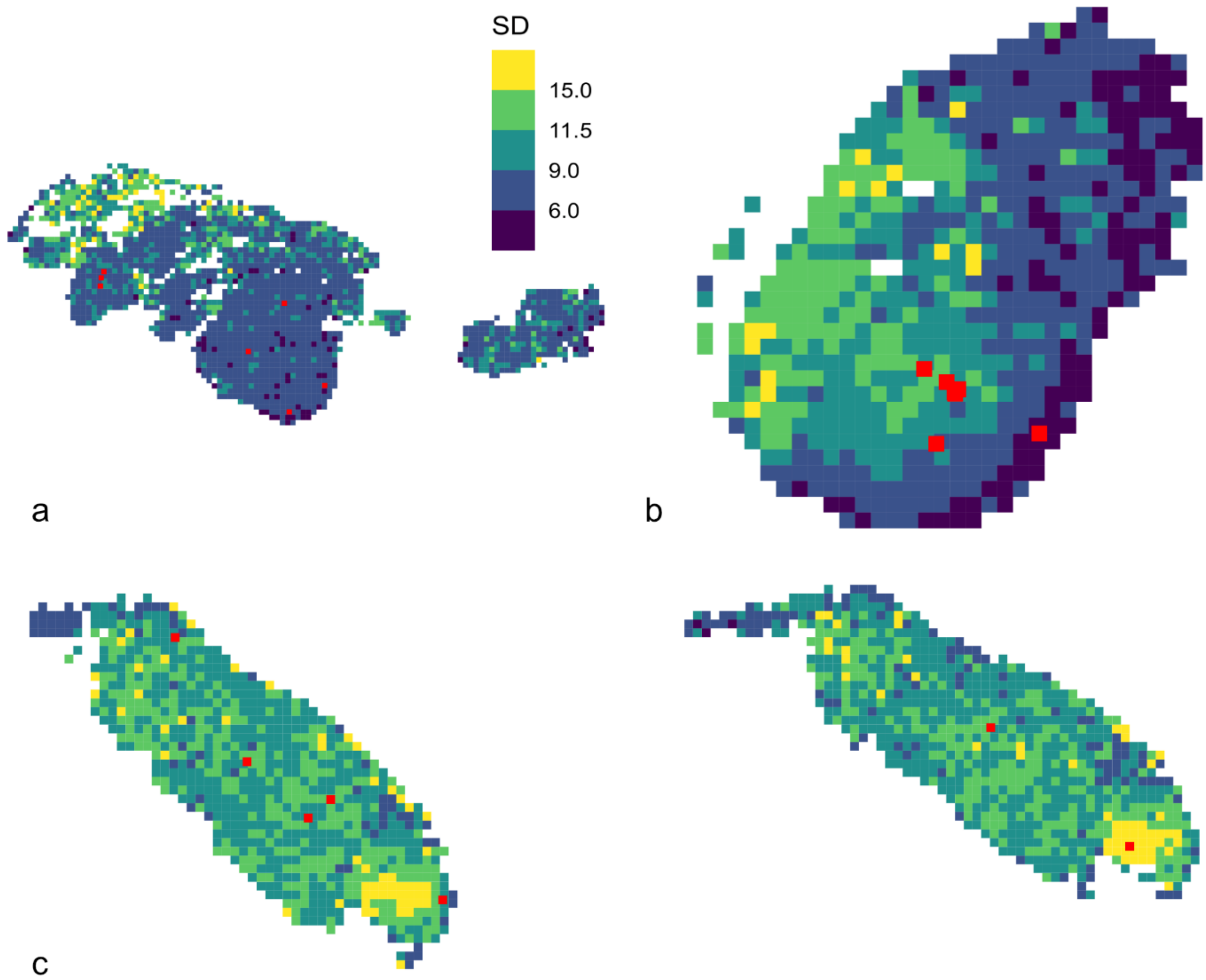

Supplemental Figure S6. Heatmaps of the anisokaryosis (standard deviation, SD, of nuclear size measured by algorithmic morphometry) from whole-slide images of canine cutaneous mast cell tumors (ccMCT) with 5 – 40% hotspot regions (SD ≥ 11.5 µm²). These patients died in relation to the ccMCT. The red patches represent the areas selected by the pathologists. (a) 11.3% hotspots, (b) 20.1% hotspots, (c) 39.7% hotspots.

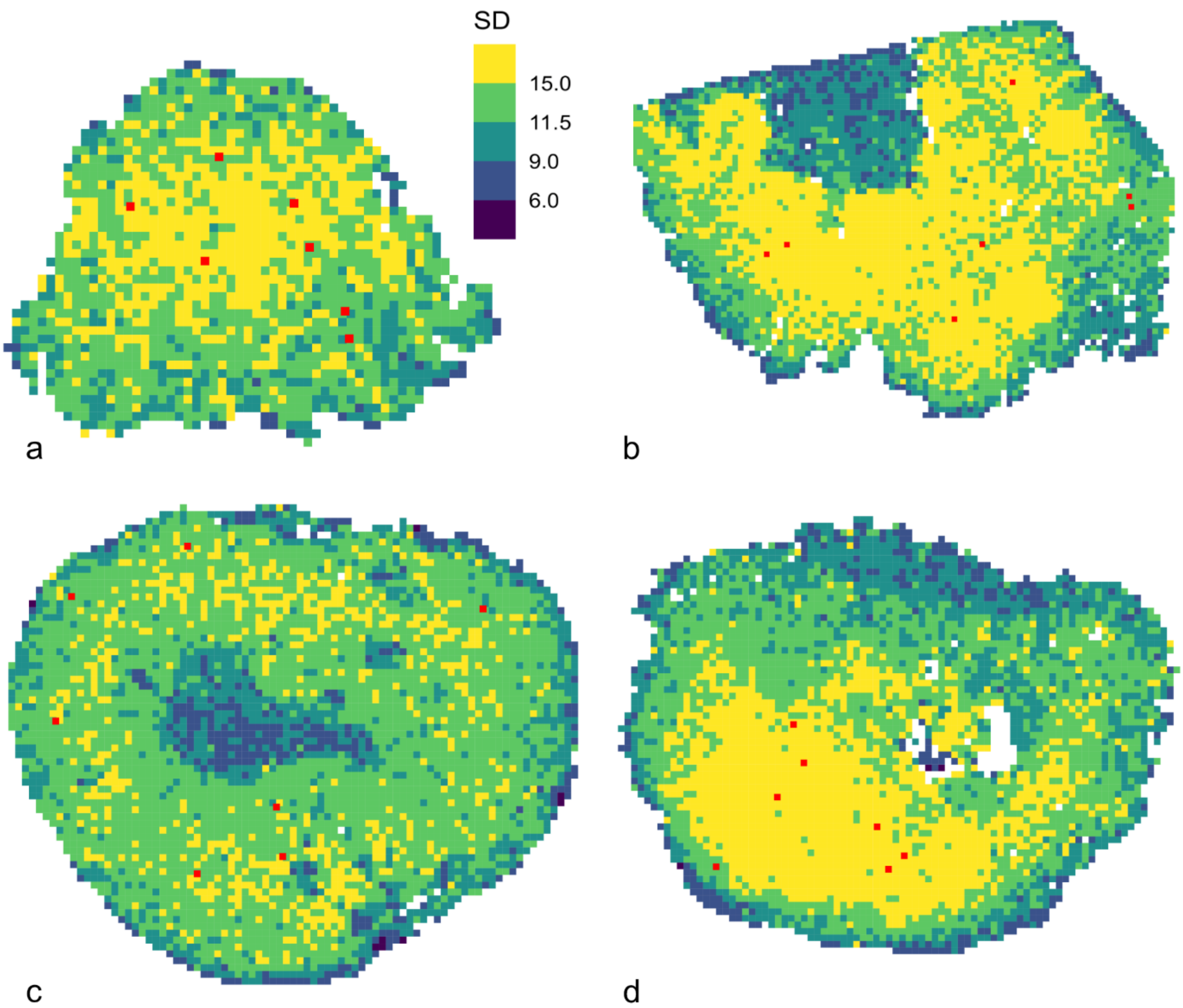

Supplemental Figure S7. Heatmaps of the anisokaryosis (standard deviation, SD, of nuclear size measured by algorithmic morphometry) from whole-slide images of canine cutaneous mast cell tumors (ccMCT) with ≥40% hotspot regions (SD ≥ 11.5 µm²). These patients died in relation to the ccMCT. The red patches represent the areas selected by the pathologists. (a) 82.1% hotspots, (b) 75.1% hotspots, (c) 74.8% hotspots, (d) 71.8% hotspots.

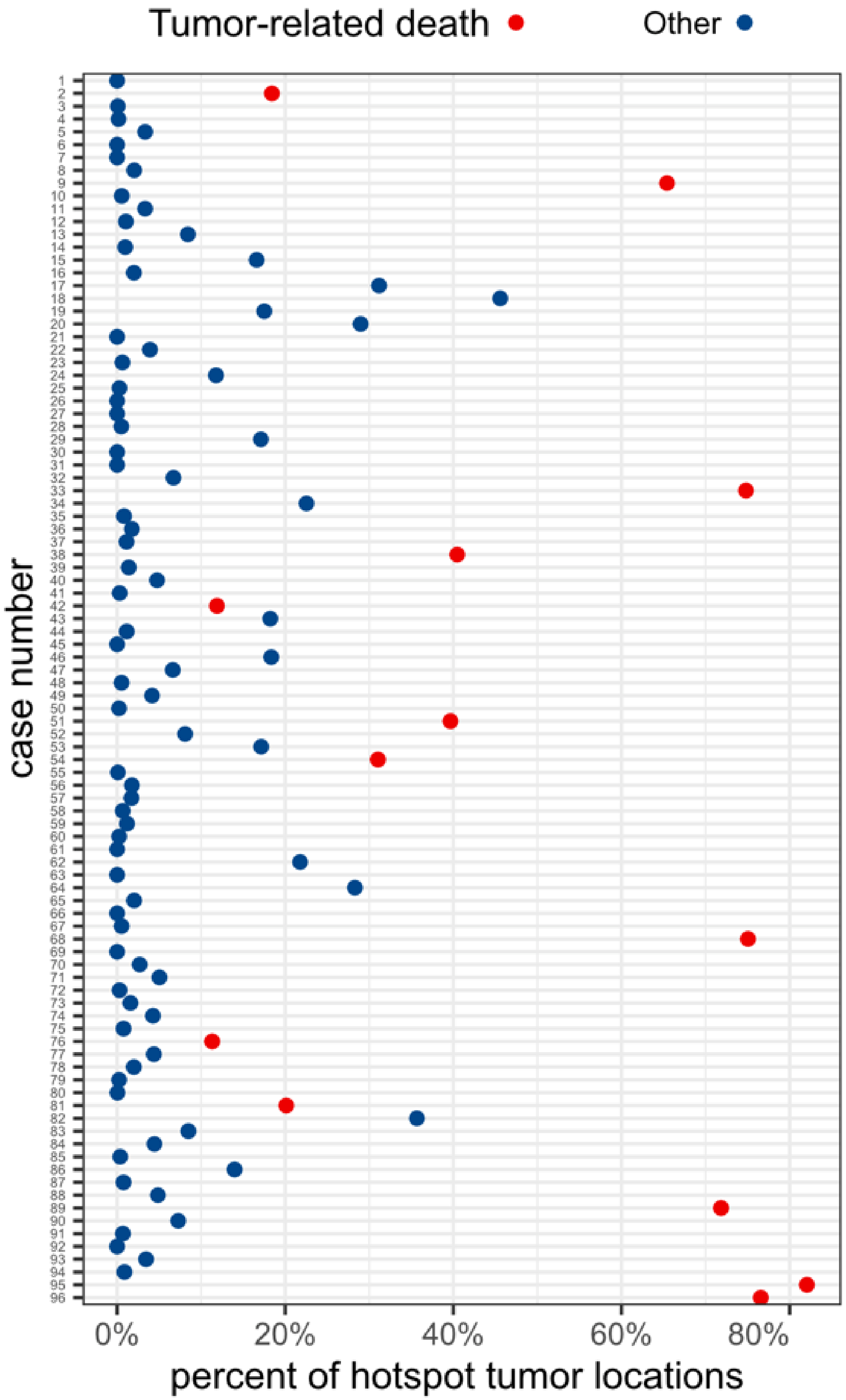

Supplemental Figure S8. Scatterplot showing the percentage of hotspot tumor regions (defined as a region with a standard deviation of nuclear size ≥ 11.5 μm²) within the entire tumor sections. Cases are categorized into tumor-related death (red dots) and other cases (blue dots) showing that most cases with a hotspot proportion of ≥20% were associated with tumor-related death.

Supplemental Table S1. Average percentile within the frequency distribution of the selected tumor regions and the associated area under the curve (AUC, tumor-related death for standard deviation of nuclear area) for the selected regions of each pathologist.

Pathologist	Average percentile and range for the selected regions	AUC
1	54.71% (1.19 – 98.89%)	0.925
2	62.88% (4.71 – 99.02%)	0.926
3	61.32% (2.26 – 98.31%)	0.925
4	34.66% (0.58 – 98.69%)	0.872
5	54.93% (0.47 – 98.11%)	0.912
6	58.26% (4.59 – 99.48%)	0.887
7	42.87% (2.24 – 99.40%)	0.883
Combined	54.58% (0.47 – 99.48%)	0.901

Supplemental Table S2. Prognostic value of the proportion of anisokaryosis hotspots (i.e., tumor region with a standard deviation of nuclear area ≥ 11.5 μm^2) depending on the threshold.

Threshold	Sensitivity	Specificity	Precision
5%	100%	73.5%	37.1%
10%	100%	81.9%	46.4%
15%	84.6%	83.1%	44.0%
20%	76.9%	91.6%	58.8%
40%	61.5%	98.8%	88.9%

Reporting guideline for manuscripts that use artificial intelligence-based automated image analysis in *Veterinary Pathology*

Version: 1.0 – brief version

Date: April 2025

Authors: Christof A. Bertram, Melissa Schutten, Lorenzo Ressel, Katharina Breininger, Joshua Webster, Marc Aubreville

Intended use of the reporting guideline:

- This checklist is a tool for authors, reviewers, and editors to verify the completeness and transparency of reporting artificial intelligence (AI)-based automated image algorithms (AIA) in manuscripts. For checklist items that are not covered in the manuscript, a short explanation why it is not applicable to the study should be added.
- The focus of this reporting guideline was placed on AI-based AIA using supervised machine learning for microscopic images.
- For this checklist, AI-based AIA is considered a measurement method that may be applied to various research topics and study types. Depending on the study topic and type, additional reporting items may be applicable.
- We encourage authors to use this 9-point checklist when preparing a manuscript on a study that has applied AI methods and to **submit this checklist as Supplemental material**, making it available to editors and reviewers.

Types of AI utilization in research studies in the field of veterinary pathology

- Application of an existing AI tool without (re)training of the AI model
    - Type 1: Application of an existing AI tool for a validated use case
    - Type 2: Application of an existing AI tool for an insufficiently validated use case without modifications of the AI tool
    - Type 3: Application of an existing AI tool with minor optimizations
- Development of new AI tool with (re)training of the AI model
    - Type 4: Development of AI tool with the same training process and the same model architecture as a previous tool
    - Type 5: Development of new AI tool with new/modified training process or model architecture

Checklist

Type of AI utilization: Type 1 (see line 91-92)

Table 1. Checklist for reporting artificial intelligence (AI)-based automated analysis (AIA) of microscopic images.

	**Items to be reported**<br>(for specifications see detailed version of the checklist)	**Type of AI utilization**	**Manuscript line numbers**<br>or explanation if not included/applicable
**Materials and Methods (M&M)**			
1	Use case / intended model output:<br>● Pathology task / pattern of interest for algorithmic evaluation<br>● Definition of the intended model output and requirements on the AI tool<br>● Suspected benefits of AIA as compared to pathologists' manual evaluation	1-5	Line 62 - 80
2	Dataset creation: datasets used for development (development dataset) and testing (test dataset) as well as application (analysis dataset) of the AI model(s)	1-5	For details on dataset creation please refer to the initial publication: https://doi.org/10.1177/03009858241295399.
2a	Short summary of the dataset(s) used for previous development and testing of the AI tool (see item 2b – d), if available	1-3	For details on the development and test dataset please refer to the initial publication: https://doi.org/10.1177/03009858241295399
2b	Cases:	2-5	For details on the cases of the development dataset, test dataset and application dataset please refer to the initial

	• Case and slide selection (inclusion and exclusion criteria)<br>• Sample characteristics: sample size (number of patients and histological sections), patient demographic information, sample origin (type of sample, organ), disease entities, sample preparation (staining procedure etc.)		publication: https://doi.org/10.1177/03009858241295399. As indicated in line 83-85, the application dataset (not used for model development) was used in this study.
2c	Image acquisition and processing:<br>• Digitization device (whole-slide image scanner type and company or camera/microscope setup)<br>• Image acquisition settings (magnification, resolution, focal planes etc.)<br>• Image processing: model input resolution (if divergent from scan resolution), WSI tiling, region of interest selection (criteria, size), normalization/standardization	2-5	Line 85-87 for the application dataset
2d	Annotations (reference standard/ground truth):<br>• Annotation method/process and rationale for selection including the annotation instructions/protocol<br>• Annotation software used: Name, version, reference (if available), company (for commercial software)<br>• Clear label class definitions and example images per class<br>• Number of annotators and level of expertise<br>• Discussion of expected sources of annotation error and biases and mitigation thereof, in	2 (test dataset)<br>3 (validation and test dataset)<br>4, 5 (training, validation, and test dataset)	Ground truth annotations were not created for this study. For details on the development and test dataset please refer to the initial publication: https://doi.org/10.1177/03009858241295399.

	particular, if semi-automated annotation was used.		
2e	Use of existing public datasets<br>● Citation of data descriptor, if available, or other reference where the data is introduced in more detail<br>● Source and date of data retrieval<br>● Short summary of the used dataset / subset (see item 2b – d)	2-5, if applicable	Not applicable
3	Dataset description:<br>● Number and distribution of cases/images and annotations per annotation class within the different data partitions (see 4a)<br>● Assessment of annotation error/biases: inter-rater agreement (if multiple annotators were involved) etc.	2-5	For details on dataset characteristics please refer to the initial publication: https://doi.org/10.1177/03009858241295399.
4	Experimental setup	2-5	Line 82-113
4a	Dataset partition procedure for algorithm development (training and validation) and testing<br>● Rationale for assumption of the development and test datasets' independence and test subset's representativeness (based on the intended use case)	3-5	This study used a previously developed algorithm. For details on model development and testing please refer to the initial publication: https://doi.org/10.1177/03009858241295399. As indicated in 83-84, the application dataset used in this study was not used for model development
4b	Methods for model performance evaluation on the test dataset and rationale for selection<br>● Statistical performance evaluation (metrics) vs. visual performance evaluation	2-5	Not applicable

	• For exclusive visual performance evaluation provide a justification why statistical evaluation is not needed<br>• Diagnostic/clinical utility measures		
4c	Quality control of model performance on the analysis dataset by visual evaluation<br><br>• Number of evaluated images and overall subjective impression	1-5, if applicable	Please refer to the initial publication: https://doi.org/10.1177/03009858241295399.
5	Development and application of algorithm	1-5	As indicated in line 91-92, the same model as described in a previous study was used. For details on model development please refer to the initial publication: https://doi.org/10.1177/03009858241295399
5a	Previously developed and commercial algorithms:<br><br>• Reference or product name and version<br>• Model architecture (if available) and summary of model development and user-defined parameters<br>• Justification of whether and why the algorithm can be applied to the analysis dataset (with or without modification)	1-3	Line 91-92<br><br>This study uses the same application dataset as the previous study (https://doi.org/10.1177/03009858241295399), which had determined a high performance on this dataset and a high prognostic discriminative ability of the algorithms measurements.
5b	Newly developed/trained algorithms:<br><br>• Design of AIA algorithms (incl. pattern recognition task of AI model, definition of input and output of the model, and automated or manual pre- and post-processing steps)<br>• Model architecture: name and reference (previously described architectures) or	4-5	Not applicable

	complete description (new/modified architectures), criteria/justification for architecture selection and modification<br>• Training process: description or reference that includes initialization of model parameters, optimization process, hyperparameters and target, data sampling strategy, regularization measures (if applicable), data imbalance mitigation (if applicable), and final model selection<br>• Software: Name, version, reference (if available), company (for commercial software)		
5c	Programming of automated pre- and post-processing steps	1-5, if applicable	As described in the initial publication: https://doi.org/10.1177/03009858241295399
5d	Application of algorithms (fully computerized vs. computer-assisted application, user interface, visualization of predictions, pre- or postprocessing etc.)<br>• Software: Name, version, reference (if available), company (for commercial software)	1-5	Fully computerized without verification/correction of model predictions, which is acknowledged as a limitation in line 205 – 206.
6	Availability of supporting data	1-5	The initial publication (https://doi.org/10.1177/03009858241295399) provides open access to supporting data.

6a	Open access to the dataset (e.g., link to repository) or reasons for withholding	1-5	The initial publication (https://doi.org/10.1177/03009858241295399) provides open access to the development and test dataset.
6b	Open access to trained model, source code along with usage notes (e.g., link to repository) or reasons for withholding	4-5	The initial publication (https://doi.org/10.1177/03009858241295399) provides open access to model code and the trained model.
**Results**			
7	Algorithmic performance evaluation	2-5	Line 92-94. For a more detailed description of model performance please refer to the initial publication: https://doi.org/10.1177/03009858241295399.
7a	Model performance (metrics) on the internal hold out test sets and, if possible, on external hold out test sets	2-5	Line 92-94. For a more detailed description of model performance please refer to the initial publication: https://doi.org/10.1177/03009858241295399.
7b	Diagnostic utility / accuracy of the algorithm	2-5	Line 157 – 159, line 171-179, figure 2
7c	Comparison to pathologists' evaluation, other state-of-the-art models, or other clinically relevant metrics.	2-5	For these experiments, please refer to the initial publication: https://doi.org/10.1177/03009858241295399.
**Discussion**			
8	Support and limitations of the AIA algorithm for the current study	1-5	Line 205-219
9	Considerations for future modifications and applications of the AIA algorithm	2-5	Not discussed in this manuscript. Please refer to the initial publication: https://doi.org/10.1177/03009858241295399.
